# Correction/Clarification about FDA Review Documents

**DOI:** 10.1371/journal.pmed.0020422

**Published:** 2005-12-27

**Authors:** Erick Turner

**Affiliations:** **1**Portland VA Medical Center and Oregon Health and Science UniversityPortland, OregonUnited States of America

Emma Veitch cites my *PLoS Medicine* Essay [1] about how the Food and Drug Administration's (FDA's) review of documents can serve as a source of clinical trials data, but she follows it up with the statement, “However, it is difficult to have confidence in data released by sponsors when the data have not been subjected to external, independent peer review. Furthermore, this information is not integrated with other data, or indexed” [2].

While I agree with the second assertion, the first assertion—that the data are not subjected to external, independent peer review—is off the mark. FDA reviews are indeed external and independent to the sponsor. These reviews are conducted not by the sponsors but by physicians and scientists employed by the United States government. True, the data originate with the sponsor. However, once the sponsor submits data to the FDA, a level of rigor and scrutiny is applied to them that is arguably higher than what occurs in the typical journal manuscript review process.

First, FDA reviewers typically revisit the original protocol submitted before the study was conducted in order to verify that the sponsor has not engaged in hypothesizing after the results are known (“HARKing”) [3]. By contrast, journal reviewers typically do not have access to the original protocol. As a result, they must trust that HARKing has not occurred, a dubious assumption in view of recent data [4].

Second, FDA statistical reviewers obtain the raw data from the sponsor, and determine whether the sponsor's findings can be replicated. By contrast, journal reviewers typically have access to only the summary statistics reported (perhaps selectively) to them by the authors or the sponsors. Consequently, reviewers can only speculate whether they could replicate the findings.

As a result, I believe that the FDA review process warrants a higher level of confidence than the conventional journal manuscript review process.
